# Space lidar observations constrain longwave cloud feedback

**DOI:** 10.1038/s41598-018-34943-1

**Published:** 2018-11-08

**Authors:** Thibault Vaillant de Guélis, Hélène Chepfer, Rodrigo Guzman, Marine Bonazzola, David M. Winker, Vincent Noel

**Affiliations:** 1 LMD/IPSL, Sorbonne Université, UPMC Univ Paris 06, CNRS, École polytechnique, Palaiseau, France; 2 LaMP/OPGC, Université Clermont Auvergne, CNRS, Clermont-Ferrand, France; 3 NASA Langley Research Center, Hampton, Virginia USA; 4 Laboratoire d’Aérologie, Université de Toulouse, CNRS, Toulouse, France

**Keywords:** Atmospheric science, Climate change

## Abstract

Some of the most challenging questions in atmospheric science relate to how clouds will respond as the climate warms. On centennial scales, the response of clouds could either weaken or enhance the warming due to greenhouse gas emissions. Here we use space lidar observations to quantify changes in cloud altitude, cover, and opacity over the oceans between 2008 and 2014, together with a climate model with a lidar simulator to also simulate these changes in the present-day climate and in a future, warmer climate. We find that the longwave cloud altitude feedback, found to be robustly positive in simulations since the early climate models and backed up by physical explanations, is not the dominant longwave feedback term in the observations, although it is in the model we have used. These results suggest that the enhanced longwave warming due to clouds might be overestimated in climate models. These results highlight the importance of developing a long-term active sensor satellite record to reduce uncertainties in cloud feedbacks and prediction of future climate.

## Introduction

As climate warms under the influence of anthropogenic radiative forcing, many climate variables are affected, some of which affect the radiative balance at the top of the atmosphere and may tend to either increase or mitigate climate warming. These feedback mechanisms make it difficult to quantify the global surface temperature increase expected at the end of century, even for a known greenhouse gas emission scenario. It is now recognized that the largest source of uncertainty in global climate model predictions is due to cloud feedbacks^1,2^. Most climate models predict a positive net cloud feedback, with shortwave (SW) and longwave (LW) cloud feedbacks both being positive^3^. The simulated positive SW cloud feedback is primarily due to a decrease in low cloud cover^4,5^. The positive LW cloud feedback is primarily due to an increase in cloud altitude^6,7^. These results from climate model simulations do not directly provide physical explanations, however, and require validation against observations. The positive LW cloud altitude feedback is generally thought to be robust as it is persistently found in climate model simulations since the very first models^3,6–13^ and is backed by a plausible physical explanation: high clouds should rise in a warming climate such that cloud temperatures remain nearly constant^13^. However, it still needs to be better verified against observations. To directly observe an altitude trend a long enough dataset is needed, highly stable in time, to detect the small changes due to anthropogenic forcing imposed on top of natural variability^14^. Spaceborne passive instrument datasets are currently the longest records available^15,16^ but have shown limited accuracy due to LW surface radiation influence through thin clouds^17–20^ and limited calibration stability over decadal timescales mainly due to calibration drifts^21,22^, which significantly increases the time required to detect climate trends. Another approach is to derive constraints on the long-term cloud feedbacks from observations of cloud natural variability on interannual scales, assuming there is a link between changes driven by natural variability and transient changes on multidecade scales^23^. Satellite active sensors (lidar, radar), have flown since 2006, provide profile observations which are highly stable in time^24^ and capable of measuring the altitude of clouds at a resolution of 30 m with long-term stability of a few meters^24^, whatever the surface type, which is much better that the existing passive remote sensing satellite instruments^17–19,24,25^. Here we propose to take advantage of seven years of space lidar observations to look for verification of the LW cloud altitude feedback mechanism in observations from the Cloud-Aerosol Lidar and Infrared Pathfinder Satellite Observation (CALIPSO) satellite^26^. The length of these lidar observations is not yet long enough to detect a climate change trend but can be used to look for a constraint on the short-term feedback from natural variability, which in turn might provide constraints on the long-term feedback.

To test the validity of the LW feedback mechanism we 1) decompose both observed and simulated LW cloud feedbacks into relative contributions due to different cloud properties, 2) evaluate the realism of the simulated short-term LW cloud feedback against observations, and 3) examine the implications for the long-term LW cloud feedback.

## Decomposing the short-term LW cloud feedback in relative contributions due to different cloud properties

**Figure 1:**
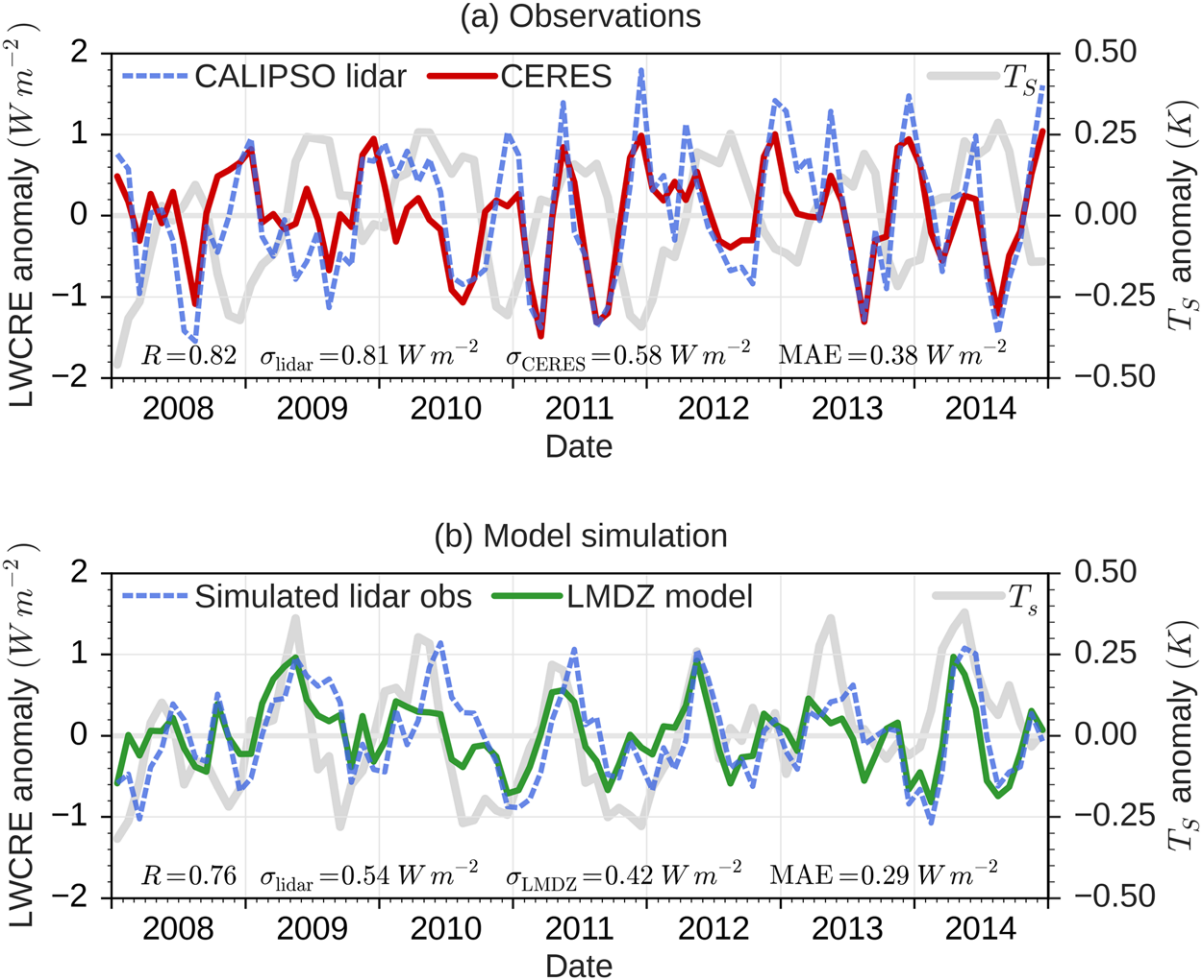
Global ocean monthly mean anomaly of the LongWave Cloud Radiative Effect (LWCRE) between January 2008 and December 2014: (**a**) observed by CERES (EBAF Ed. 4.0) and derived from space lidar observations, (**b**) simulated by the LMDZ general circulation model and derived from synthetic space lidar observations obtained with a lidar simulator plugged on the LMDZ model. The anomaly is the difference between the value of a month and the mean over the whole 2008–2014 period. Surface temperature anomaly from ERA-I for observations and from model output for simulations are shown in light gray. Coefficient correlation *R*, standard deviations *σ*, and Mean Absolute Error (MAE) are given at the bottom of subplots.

The LW cloud feedback can be expressed as the change in the LW cloud radiative effect (LWCRE) at the top-of-atmosphere (TOA) by a degree change of surface temperature. To decompose the short-term LW cloud feedback into relative contributions from different cloud variables, we first need to verify that we can retrieve the LWCRE from lidar observations. Space lidar provide accurate observations of the five following cloud properties which are linearly linked to the LWCRE^27^: the opaque cloud altitude ${Z}_{{T}_{{\rm{Opaque}}}}$, the optically thin cloud altitude ${Z}_{{T}_{{\rm{Thin}}}}$, the opaque cloud cover ${C}_{{\rm{Opaque}}}$, the optically thin cloud cover ${C}_{{\rm{Thin}}}$, and the thin cloud emissivity ${\varepsilon }_{{\rm{Thin}}}$. The LWCRE can be retrieved from these five cloud properties (Method). It has been shown that opaque clouds play a fundamental role in the LWCRE amplitude^28,29^ and variations^27^. Figure 1a compares the LWCRE variations retrieved from the space lidar with the one derived from observations of broadband LW TOA fluxes by the Clouds and the Earth’s Radiant Energy System (CERES) radiometers^30,31^. The LWCRE variations retrieved from the lidar are seen to be consistent with the radiometer observations (R = 0.82), the mean absolute error is half of the CERES standard deviation, and monthly mean differences between CERES LWCRE and LWCRE retrieved from the lidar falls within the CERES global mean nighttime LW flux uncertainty (2.4 W m^−2^)^31^.

Figure 1b shows the counterpart in climate model simulations. We evaluate the LWCRE retrieved from a virtual lidar overflying the atmosphere simulated by a climate model against the LWCRE computed from model TOA fluxes. A spaceborne lidar simulator^32,33^ allows the five synthetic cloud properties defined above to be derived consistently from the model and from the observations. The LWCRE is then retrieved from the five synthetic cloud properties. Figure 1b compares LWCRE derived from the virtual lidar implemented within the LMDZ model^34^ with the LWCRE obtained directly from the LMDZ model fluxes^35^. We see again a good estimate (R = 0.76) of the LWCRE variations derived from the five synthetic lidar cloud properties compared to the more detailed radiative transfer computation results given by the model.

We also plot on Fig. 1a the global mean surface temperature from ERA-Interim reanalysis^36^ and on Fig. 1b the global mean surface temperature from the model outputs. Even if this present-day climate simulation (AMIP) is forced by observed sea surface temperatures^37^, we note the surface temperature anomalies are not exactly the same in reanalysis and simulations. This is due to differences in surface temperature of sea ice. However it does not explain the differences between the observed and simulated LWCRE as we note the variations of the simulated LWCRE is mostly in phase with the variations of the surface temperature whereas it is mostly the opposite in observations. It suggests that the simulation of change in cloud properties due to surface temperature anomaly is not well represented in the model.

Results shown in Fig. 1 suggest that the lidar observations, real or simulated, can be used to infer the LWCRE variations with sufficient accuracy to decompose the short-term LW cloud feedback in relative contributions due to cloud properties.

Next, we estimate the so-called “short-term cloud feedback”^38,39^. In the observations, we regress the observed global monthly mean LWCRE anomalies against the global monthly mean surface temperature anomalies from reanalysis during the time period 2008–2014. Then we decompose this observed short-term LW cloud feedback into five cloud property contributions thanks to simple relationships between the properties and the LWCRE^27,28^ (Methods). In the model simulations, we follow the same method as with the observations to estimate the simulated short-term LW cloud feedback and its five components using the LWCRE, the surface temperature and the five cloud properties simulated during the period 2008–2014. In addition to the simulated short-term cloud feedback, we also compute the simulated long-term cloud feedback using present-day climate simulations (AMIP) and future climate simulations for which surface temperature is artificially increased by 4 K (AMIP + 4 K) (Methods).

**Figure 2:**
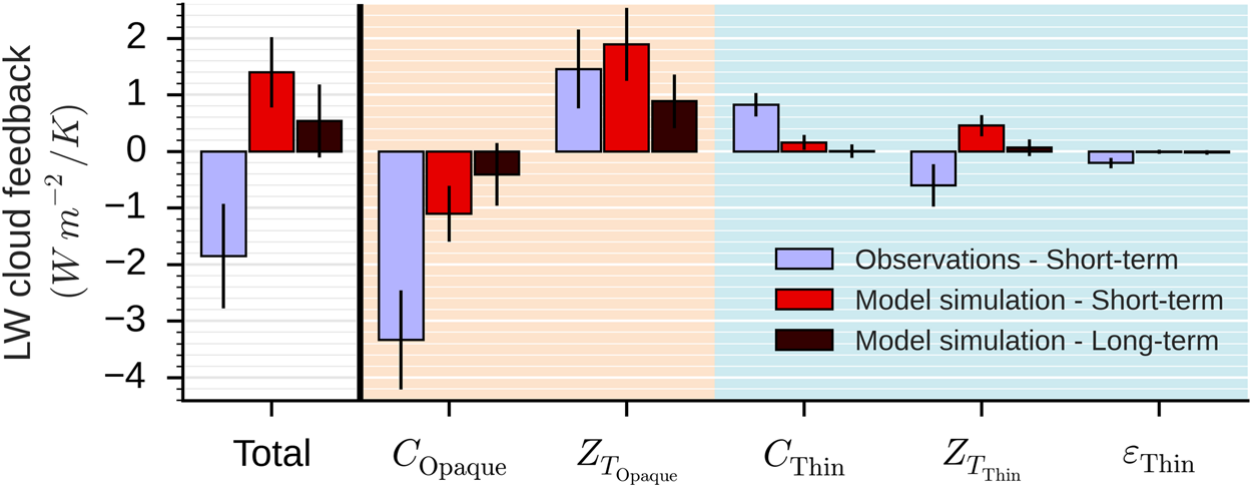
Decomposition of the longwave cloud feedback into five components: the cover of opaque clouds $({C}_{{\rm{Opaque}}})$, the altitude of the opaque clouds $({Z}_{{T}_{{\rm{Opaque}}}})$, the cover of thin clouds $({C}_{{\rm{Thin}}})$, the altitude of thin clouds $({Z}_{{T}_{{\rm{Thin}}}})$, the emissivity of thin clouds $({\varepsilon }_{{\rm{Thin}}})$. The observed short-term (blue) is derived from space lidar data between 2008 and 2014. The simulated short-term (red) is derived from model + lidar simulator simulation in present-day climate (AMIP) between 2008 and 2014. The simulated long-term (dark red) is derived from model simulations in present-day climate (AMIP) and in a warmer future climate (AMIP + 4 K). All the results are based on monthly mean data over global ocean. Lines on bars are the 95% confidence interval.

Figure 2 shows the short-term LW cloud feedback derived from observations (blue), the short-term LW cloud feedback derived from the present-day climate simulation (red), and the long-term LW cloud feedback derived from the change between the future and the present-day climate simulations (dark red).

## Evaluation of the simulated short-term LW cloud feedback against the observation between 2008 and 2014

We apply the same lidar-based framework to observations and the model to consistently compare the observed and simulated short-term cloud feedbacks. Figure 2 shows substantial differences between the observed and simulated feedbacks. Unlike the simulations, the observed short-term LW cloud feedback is negative. It is a consequence of the anti-correlation between variations of surface temperature and LWCRE in the observations while there is a correlation between those variables in the present-day simulation (Fig. 1). The simulated cloud feedback in the model is not correct because the model does not correctly reproduce cloud changes associated to surface temperature change. If we look at the decomposition of the total cloud feedback in cloud properties, we notice that the observed short-term LW cloud feedback is mainly driven by the opaque cloud cover variations between 2008 and 2014. Variations of the opaque cloud altitude do not play the dominant role for the observations, while it is the dominant component in the model. In fact, the model is able to reproduce the observed amplitude of the variations in the opaque cloud altitude, but significantly underestimates the observed amplitude of the variations in the opaque cloud cover. First investigations of this model underestimation suggest a lack from model to simulate cloud opacity interannual variations which transfer part of cloud from opaque cloud cover to thin cloud cover and conversely.

## Implication for the long-term LW cloud feedback

Obviously, the long-term cloud feedback cannot be observed now. However, there are some possibilities for the long-term cloud feedback mechanism associated to climate warming to be linked to the short-term feedback associated to present-day climate variations^23,39–41^. Under the assumption there is a relationship between simulated present day and transient scale variabilities, observed relationships between clouds and their controlling factors can be used to constrain long-term feedbacks from models. Given that the length of the CALIPSO record has a limited number of years, and relying on a recent study^41^ which suggests that there maybe a relationship for some climate feedbacks between feedback strength at monthly and transient timescales, we estimated the short-term feedback from monthly mean data.

Figure 2 shows that the simulated long-term LW cloud feedback has the same sign as the simulated short-term feedback but with smaller amplitudes. It is consistent with the study of Zhou *et al*.^42^. The simulated short-term and long-term LW cloud feedbacks are both positive, and mainly due to the change in opaque cloud altitude, although partially compensated by the change in opaque cloud cover. The thin cloud properties do not play a significant role.

The similarities between short-term and long-term simulated LW cloud feedbacks suggest there is a link between the two in the model world. Supposing such a link also exists in the real world, then comparison of the simulated and observed short-term LW cloud feedbacks can provide a constraint on the long-term LW cloud feedback. Results shown in Fig. 2 suggest that the long-term positive LW cloud altitude feedback mechanism —robustly reproduced by all climate models for nearly five decades— is not the dominant term, at least over the oceans. Uncertainty in amplitude of the positive LW cloud altitude feedback simulated by model is large^3^ and the basic cloud-rise mechanism may be complicated by factors such a possible decrease in tropical anvil coverage which tends to accompany the cloud rise according to a “stability-iris” mechanism^43^.

When considering the total cloud feedback (LW + SW), the opaque cloud cover change feedback (which dominates the negative LW feedback over oceans) has likely less impact because its effect on longwave radiation partially compensates its positive feedback effect on the shortwave radiation.

## Conclusion

Recent work shows a large inter-model spread in cloud feedbacks^3^. These feedbacks are poorly constrained by traditional satellite records because they do not provide robust and stable measurements of the cloud vertical profile independently, of the surface and independently, of the cloud opacity. As shown herein, active remote sensor records like the lidar on board CALIPSO or the lidar and radar on board the future Earth Clouds, Aerosol and Radiation Explorer (EarthCARE)^44^ satellite are powerful new tools to constrain the cloud feedback^45,46^ and, ultimately, reduce uncertainties on climate sensitivity estimates.

## Methods

In the main text of this paper, we determine the relative contribution of five different cloud properties derived from space lidar observations^28^ to the LW cloud feedback (Fig. 2). The summation of the five contributions equal to the total contribution. Here, we describe the successive steps to determine these relative contributions: (1) we establish a linear relationship linking the LWCRE to the five cloud properties, (2) we decompose the LWCRE temporal variations into contributions due to the five cloud properties variations, (3) we determine the relative contribution of each cloud property to the short-term LW cloud feedback, and (4) we determine the relative contribution of each cloud property to the long-term LW cloud feedback. This method is applied both for real and simulated space lidar observations.

### Linear relationship between the LWCRE and the five cloud properties

The linear relationship between the LWCRE and the altitude of the opaque cloud temperature $({Z}_{{T}_{{\rm{Opaque}}}})$, the altitude of the thin cloud temperature $({Z}_{{T}_{{\rm{Thin}}}})$, the opaque cloud cover $({C}_{{\rm{Opaque}}})$, the thin cloud cover $({C}_{{\rm{Thin}}})$, and thin cloud emissivity $({\varepsilon }_{{\rm{Thin}}})$ is:

1 $${{\rm{LWCRE}}}_{{\rm{Total}}}=2{\rm{\Gamma }}{C}_{{\rm{Opaque}}}{Z}_{{T}_{{\rm{Opaque}}}}+2{\rm{\Gamma }}{C}_{{\rm{Thin}}}{{\rm{\varepsilon }}}_{{\rm{Thin}}}{Z}_{{T}_{{\rm{Thin}}}},$$

where the first term and second term on the right hand side of the equation represent respectively the opaque cloud contribution and the thin cloud contribution to the LWCRE. ${\rm{\Gamma }}=\frac{-dT}{dz}$ is the mean temperature lapse rate in the troposphere (annual global mean over ocean from ERA-I reanalysis is ${\rm{\Gamma }}=5.5$ K km^−1^) and the unit of the scalar constant “2” is W m^−2^ K^−1^.

First, we establish Eq. (1) analytically. A previous study^28^ defined the opaque and thin cloud temperatures (${T}_{{\rm{Opaque}}}$ and ${T}_{{\rm{Thin}}}$ expressed in K) and found a linear relationship between these temperatures and the LWCRE:

2 $${{\rm{LWCRE}}}_{{\rm{Total}}}={C}_{{\rm{Opaque}}}({{\rm{OLR}}}_{{\rm{Clear}}}-(2{T}_{{\rm{Opaque}}}-310))+{C}_{{\rm{Thin}}}{{\rm{\varepsilon }}}_{{\rm{Thin}}}({{\rm{OLR}}}_{{\rm{Clear}}}-(2{T}_{{\rm{Thin}}}-310)),$$

where ${{\rm{OLR}}}_{{\rm{Clear}}}$ is the outgoing LW radiation in clear-sky conditions and the unit of the scalar constant “2” is W m^−2^ K^−1^ and “310” is W m^−2^.

Assuming the temperature lapse rate Γ is constant throughout the troposphere, ${Z}_{{T}_{{\rm{Opaque}}}}$ can be written as: ${Z}_{{T}_{{\rm{Opaque}}}}=1/{\rm{\Gamma }}({T}_{s}-{T}_{{\rm{Opaque}}})$ where *T*_*s*_ is the surface temperature. Similarly ${Z}_{{T}_{{\rm{Thin}}}}$ can be expressed as a function of ${T}_{{\rm{Thin}}}$ and *T*_*s*_. Assuming the surface can be considered as an opaque cloud at the temperature *T*_*s*_, Eq. (1) can be derived from Eq. (2) as follows:

3 $$\begin{array}{rcl}{{\rm{LWCRE}}}_{{\rm{Total}}} & = & {C}_{{\rm{Opaque}}}[(2{T}_{s}-310)-(2{T}_{{\rm{Opaque}}}-310)]\\  &  & +\,{C}_{{\rm{Thin}}}{{\rm{\varepsilon }}}_{{\rm{Thin}}}[(2{T}_{s}-310)-(2{T}_{{\rm{Thin}}}-310)]\\  & = & {C}_{{\rm{Opaque}}}2({T}_{s}-{T}_{{\rm{Opaque}}})+{C}_{{\rm{Thin}}}{{\rm{\varepsilon }}}_{{\rm{Thin}}}2({T}_{s}-{T}_{{\rm{Thin}}})\\  & = & {C}_{{\rm{Opaque}}}2{\rm{\Gamma }}{Z}_{{T}_{{\rm{Opaque}}}}+{C}_{{\rm{Thin}}}{{\rm{\varepsilon }}}_{{\rm{Thin}}}2{\rm{\Gamma }}{Z}_{{T}_{{\rm{Thin}}}}\end{array},$$

**Figure 3:**
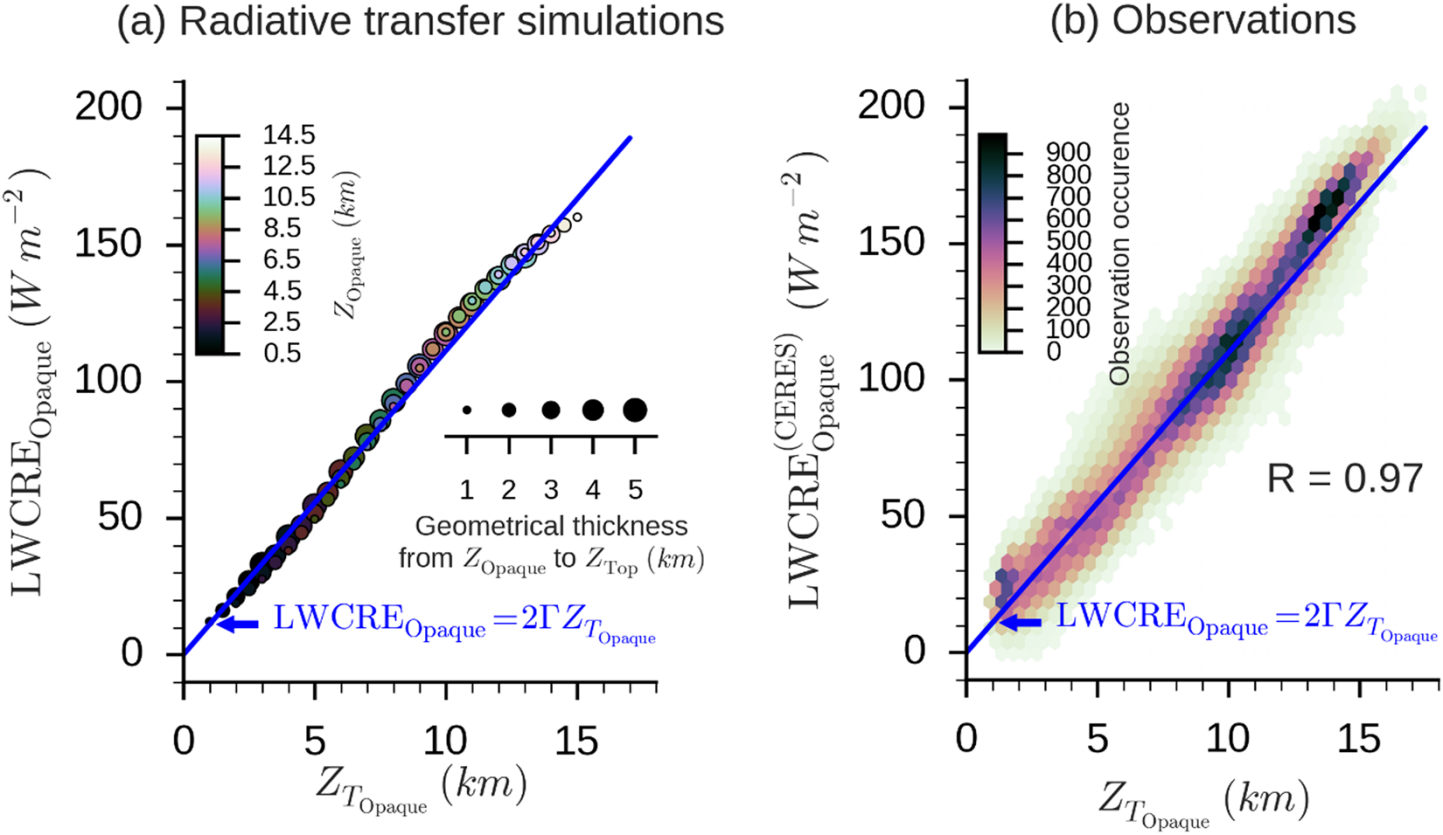
(**a**) Radiative transfer simulations of the LongWave Cloud Radiative Effect (LWCRE) for an atmospheric single column containing an opaque cloud moving in altitude (each dot represents the result for one computation). The color of dots represents the altitude where the lidar ends fully attenuated into the opaque cloud ${Z}_{{\rm{Opaque}}}$ (0.5 km [dark] – 14.5 km [bright]) and the size of dots the geometrical thickness from ${Z}_{{\rm{Opaque}}}$ to cloud top ${Z}_{{\rm{Top}}}$ (1 km [small] – 5 km [large]). There is a clear linear relationship between LWCRE and the cloud altitude ${Z}_{{T}_{{\rm{Opaque}}}}=({Z}_{{\rm{Opaque}}}+{Z}_{{\rm{Top}}})/2$. Results shown here use the year 2008 mean thermodynamic atmospheric variables over the oceans from ERA-I reanalysis. (**b**) LWCRE derived from CERES radiometer observations as a function of ${Z}_{{T}_{{\rm{Opaque}}}}$ measured by collocated CALIPSO space-lidar over oceans over 2008–2010. ${\rm{\Gamma }}=\frac{-dT}{dz}$ is the annual global mean temperature lapse rate in the troposphere over ocean from ERA-I reanalysis.

Next, we evaluate the validity of Eq. (1) against 1) results from detailed radiative transfer computations and 2) collocated observations of the CALIPSO space-lidar and the CERES radiometer^47^. The radiative transfer computations were performed with the GAME radiative transfer code^48^ for an atmosphere with an overcast opaque cloud (${C}_{{\rm{Opaque}}}=1$) located at various altitudes from the surface to the tropopause. Observations used are all single-layer opaque cloud sounded by the space-lidar over oceans over 2008–2010. Figures 3a,b show the relationship between the LWCRE and ${Z}_{{T}_{{\rm{Opaque}}}}$ from the radiative transfer code and in observations.

The detailed radiative transfer computations (Fig. 3a) and observations (Fig. 3b) confirm the linear dependence of ${{\rm{LWCRE}}}_{{\rm{Opaque}}}$ on ${Z}_{{T}_{{\rm{Opaque}}}}$. The blue line represents the analytical relationship of Eq. (1). A previous study^49^ obtained a similar relationship and showed that it does not depend on the region considered. Therefore, above an opaque cloud, the LWCRE increases by 11 W m^−2^ when the altitude of the opaque cloud rises by 1 km. We performed a similar analysis for the thin clouds, using as well direct radiative transfer computations and observations (CALIPSO and CERES). It shows that the ${{\rm{LWCRE}}}_{{\rm{Thin}}}$ depends linearly on the product ${\varepsilon }_{{\rm{Thin}}}{Z}_{{T}_{{\rm{Thin}}}}$. The emissivity varies between 0 and 0.8, and is inferred from CALIPSO. Here after we use the relationship:

4 $${{\rm{LWCRE}}}_{{\rm{Total}}}=11({C}_{{\rm{Opaque}}}{Z}_{{T}_{{\rm{Opaque}}}}+{C}_{{\rm{Thin}}}{{\rm{\varepsilon }}}_{{\rm{Thin}}}{Z}_{{T}_{{\rm{Thin}}}})$$

### Decomposition of the LWCRE temporal variations into contributions due to cloud property variations

As describe in a previous study^27^, the change in ${{\rm{LWCRE}}}_{{\rm{Total}}}$ between two states of the atmosphere, ${t}_{1}$ and ${t}_{2}$, can be written:

5 $${{\rm{\Delta }}\mathrm{LWCRE}}_{{\rm{Total}}}={{\rm{\Delta }}\mathrm{LWCRE}}_{{\rm{Opaque}}}+{{\rm{\Delta }}\mathrm{LWCRE}}_{{\rm{Thin}}},$$

where ${\rm{\Delta }}$ indicates the change between ${t}_{1}$ and ${t}_{2}$.

The opaque term ${{\rm{\Delta }}\mathrm{LWCRE}}_{{\rm{Opaque}}}$ can be expressed as a sum of changes due to ${C}_{{\rm{Opaque}}}$ and ${Z}_{{T}_{{\rm{Opaque}}}}$:

6 $${{\rm{\Delta }}\mathrm{LWCRE}}_{{\rm{Opaque}}}=\frac{\partial {{\rm{LWCRE}}}_{{\rm{Opaque}}}}{\partial {C}_{{\rm{Opaque}}}}{\rm{\Delta }}{C}_{{\rm{Opaque}}}+\frac{\partial {{\rm{LWCRE}}}_{{\rm{Opaque}}}}{\partial {Z}_{{T}_{{\rm{Opaque}}}}}{\rm{\Delta }}{Z}_{{T}_{{\rm{Opaque}}}},$$

where the two derivatives are obtained from Eq. (4) and estimated at the mean of the two states $\frac{{t}_{1}+{t}_{2}}{2}$ in order to cancel out residual nonlinear term^27,50^.

Similarly, the thin term can be expressed as a sum of changes due to ${C}_{{\rm{Thin}}}$, ${Z}_{{T}_{{\rm{Thin}}}}$, and ${\varepsilon }_{{\rm{Thin}}}$:

7 $${{\rm{\Delta }}\mathrm{LWCRE}}_{{\rm{Thin}}}=\frac{\partial {{\rm{LWCRE}}}_{{\rm{Thin}}}}{\partial {C}_{{\rm{Thin}}}}{\rm{\Delta }}{C}_{{\rm{Thin}}}+\frac{\partial {{\rm{LWCRE}}}_{{\rm{Thin}}}}{\partial {Z}_{{T}_{{\rm{Thin}}}}}{\rm{\Delta }}{Z}_{{T}_{{\rm{Thin}}}}+\frac{\partial {{\rm{LWCRE}}}_{{\rm{Thin}}}}{\partial {{\rm{\varepsilon }}}_{{\rm{Thin}}}}{\rm{\Delta }}{\varepsilon }_{{\rm{Thin}}}+\mathrm{NL},$$

where the three derivatives are obtained from Eq. (4) and estimated at the mean of the two states $\frac{{t}_{1}+{t}_{2}}{2}$. In this last equation, a non-linear residual term remains but is negligible^27^.

### Determining the relative contribution of each cloud property to the short-term LW cloud feedback

**Figure 4:**
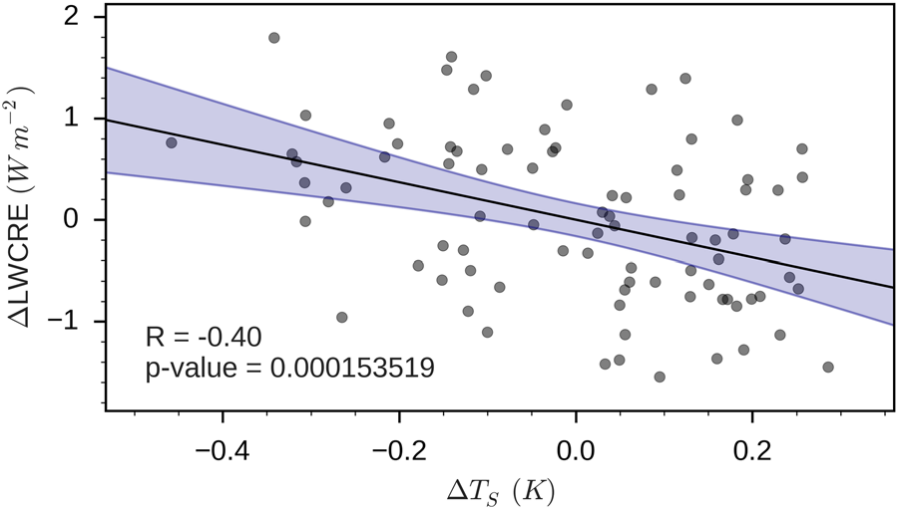
Scatter plot of the 84 monthly global mean LongWave Cloud Radiative Effect (LWCRE) anomalies over ocean derived from spaceborne lidar observations over 2008–2014 versus monthly global mean surface temperature anomalies. The solid line is the linear least-squares fit. Blue shading denote the 95% confidence interval of the fit.

The short-term cloud feedback is computed from the natural climate variability in the present-day climate. To estimate the relative contribution of a cloud property (e.g. ${C}_{{\rm{Opaque}}}$) to the short-term LW cloud feedback, we regress its global monthly mean relative contributions as defined in Eqs. (5–7) (e.g. $\frac{\partial {{\rm{LWCRE}}}_{{\rm{Opaque}}}}{\partial {C}_{{\rm{Opaque}}}}{\rm{\Delta }}{C}_{{\rm{Opaque}}}$) against the global monthly mean surface temperature anomaly ${\rm{\Delta }}{T}_{S}$ over the 2008–2014 time period. Uncertainties are defined as the 95% confidence interval of the regression. Figure 4 shows the regression of the global monthly mean total LWCRE anomaly against the global monthly mean surface temperature anomaly in observations with its 95% confidence interval. It suggests that cloud feedback is negative over this time period with a value of −1.85 ± 0.93 W m^−2^ K^−1^ (reported in first bar of Fig. 2).

We estimate the relative contribution of each cloud property to the short-term LW cloud feedback independently in both observations and the model. In the observations, the five cloud properties come from the GCM-Oriented CALIPSO Cloud Product (CALIPSO-GOCCP) dataset^29,51^ between 2008 and 2014. Observations before 2008 are not considered because the CALIPSO lidar view angle was changed from 0.3 to 3° in November 2007, so the cloud properties observed before November 2007 are not fully consistent with those observed after. In the model, the cloud properties come from an Atmospheric Model Intercomparison Project (AMIP) simulation^52^ which is forced by monthly mean surface temperature between 2008 and 2014 from ERA-Interim reanalysis^38^. The AMIP simulation is performed using the CFMIP Observation Simulator Package (COSP)^32^ lidar simulator^29,33,53^, so that the five cloud properties output from the model simulation are fully consistent with the observed five cloud properties in the GOCCP dataset.

### Determining the relative contribution of each cloud property to the long-term LW cloud feedback

The long-term cloud feedback is due to the forced warming of climate over several decades. To estimate the relative contribution of a cloud property to the future long-term LW cloud feedback, we necessarily rely only on climate simulations: a simulation of the current climate and a future climate simulation. The long-term LW cloud feedback is estimated by dividing the global mean LWCRE change between the future and the present-day climate (${\rm{\Delta }}\mathrm{LWCRE}$) by the global mean surface temperature change between the present-day and future climate (${\rm{\Delta }}{T}_{S}$). The long-term LW cloud feedback is estimated for each month independently (e.g. “March 2009 + 4 K” - “March 2009”), and then averaged over all 84 months available. The uncertainty is the standard deviation over the 84 months.

The present-day climate simulation is the same as in the previous section (3): a simulation forced by ERA-Interim sea surface temperature between 2008 and 2014 (AMIP simulation type). The future climate simulation is forced by ERA-Interim sea surface temperature between 2008 and 2014 to which we artificially add 4 K (“AMIP + 4 K” simulation type).

## Data Availability

The CALIPSO-GOCCP dataset is available online at http://climserv.ipsl.polytechnique.fr/cfmip-obs/. The cloud diagnostics used with the LMDZ simulations will be available in COSP v2.
