# Author Correction: Space lidar observations constrain longwave cloud feedback

**DOI:** 10.1038/s41598-020-74323-2

**Published:** 2020-10-07

**Authors:** Thibault Vaillant de Guélis, Hélène Chepfer, Rodrigo Guzman, Marine Bonazzola, David M. Winker, Vincent Noel

**Affiliations:** 1 LMD/IPSL, Sorbonne Université, UPMC Univ Paris 06, CNRS, École polytechnique, Palaiseau, France; 2 LaMP/OPGC, Université Clermont Auvergne, CNRS, Clermont-Ferrand, France; 3 NASA Langley Research Center, Hampton, Virginia USA; 4 Laboratoire d’Aérologie, Université de Toulouse, CNRS, Toulouse, France

Correction to: *Scientific Reports* 10.1038/s41598-018-34943-1, published online 08 November 2018

This Article contains an error in Figure 2 where the uncertainty values for the long-term model simulation are incorrect.

The correct Figure 2 appears below as Figure 1. Table 1 contains the corresponding feedback values.

**Figure 1:**
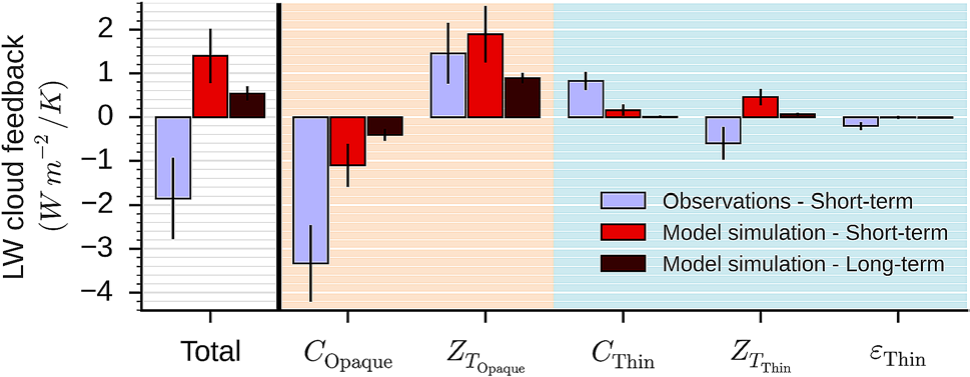
Decomposition of the longwave cloud feedback into five components: the cover of opaque clouds (*C*_Opaque_), the altitude of the opaque clouds ($Z_{{T_{{{\text{Opaque}}}} }}$), the cover of thin clouds (*C*_Thin_), the altitude of thin clouds ($Z_{{T_{{{\text{Thin}}}} }}$), the emissivity of thin clouds (*ε*_Thin_). The observed short-term (blue) is derived from space lidar data between 2008 and 2014. The simulated short-term (red) is derived from model + lidar simulator simulation in present-day climate (AMIP) between 2008 and 2014. The simulated long-term (dark red) is derived from model simulations in present-day climate (AMIP) and in a warmer future climate (AMIP + 4 K). All the results are based on monthly mean data over global ocean. Lines on bars are the 95% confidence interval.

**Table 1:** Cloud feedback values shown in Fig. 2 with the 95% confidence interval in parenthesis.

	Total	C_Opaque	Z_T_Opaque	C_Thin	Z_T_Thin	E_Thin
Obs short-term	− 1.85 (± 0.93)	− 3.33 (± 0.88)	+ 1.46 (± 0.70)	+ 0.83 (± 0.21)	− 0.60 (± 0.37)	− 0.20 (± 0.09)
Simu short-term	+ 1.40 (± 0.62)	− 1.10 (± 0.49)	+ 1.89 (± 0.64)	+ 0.16 (± 0.13)	+ 0.46 (± 0.18)	− 0.01 (± 0.04)
Simu long-term	+ 0.54 (± 0.16)	− 0.41 (± 0.14)	+ 0.89 (± 0.12)	+ 0.01 (± 0.03)	+ 0.07 (± 0.04)	− 0.02 (± 0.01)

